# Triggering comprehensive enhancement in oxygen evolution reaction by using newly created solvent

**DOI:** 10.1038/srep28456

**Published:** 2016-06-22

**Authors:** Hsiao-Chien Chen, Fu-Der Mai, Kuang-Hsuan Yang, Liang-Yih Chen, Chih-Ping Yang, Yu-Chuan Liu

**Affiliations:** 1 Department of Biochemistry and Molecular Cell Biology, School of Medicine, College of Medicine, Taipei Medical University, No. 250, Wuxing St., Taipei 11031, Taiwan; 2 Department of Materials Science and Engineering, Vanung University, No. 1, Van Nung Rd., Chung-Li City, Taiwan; 3 Department of Chemical Engineering, National Taiwan University of Science and Technology, No. 43, Sec. 4, Keelung Rd., Taipei 10607, Taiwan; 4 Graduate Institute of Medical Science, College of Medicine, Taipei Medical University, No. 250, Wuxing St., Taipei 11031, Taiwan

## Abstract

Theoretical calculations indicate that the properties of confined liquid water, or liquid water at surfaces, are dramatically different from those of liquid bulk water. Here we present an experimentally innovative strategy on comprehensively efficient oxygen evolution reaction (OER) utilizing plasmon-induced activated water, creating from hot electron decay at resonantly illuminated Au nanoparticles (NPs). Compared to conventional deionized (DI) water, the created water owns intrinsically reduced hydrogen-bonded structure and a higher chemical potential. The created water takes an advantage in OER because the corresponding activation energy can be effectively reduced by itself. Compared to DI water-based solutions, the OER efficiencies at Pt electrodes increased by 69.3%, 21.1% and 14.5% in created water-based acidic, neutral and alkaline electrolyte solutions, respectively. The created water was also effective for OERs in photoelectrochemically catalytic and in inert systems. In addition, the efficiency of OER increased by 47.5% in created water-based alkaline electrolyte solution prepared *in situ* on a roughened Au electrode. These results suggest that the created water has emerged as an innovative activator in comprehensively effective OERs.

A unique feature of liquid water is its highly labile state with a network of hydrogen bonds (HBs), in which the breaking and reforming of HBs occur at a picosecond timescale^1^,^2^. Most physical and chemical processes in aqueous solutions, in which reactants are surrounded by ultrafast motions of liquid water, are dependent on HBs^3^,^4^. HBs of liquid water have the dual functions of acting on the water itself to form water clusters and also interacting with other species. The strength when acting on itself determines the size of water clusters; while the availability of interactions with other species is relative to the activity of the liquid water. Theoretically, water deviates from the tetrahedral symmetry observed in bulk water, creating disordered defects that reduce the scale of water clusters. However, directly quantitatively measuring the exact scale of water clusters in different states remains challenging. Compared to bulk bound water clusters, disordered water clusters with weak HBs have more free water molecules which can interact with other species to enhance the activity. On the other hand, the oxygen evolution reaction (OER) is an oxidation process for producing oxygen by electrolytically splitting water, which represents an efficient and green technology for energy conversion and storage^5^,^6^,^7^. To increase the efficiency of OERs, the most common approaches have focused on new and cheap catalysts with different chemical compositions and structures^8^,^9^,^10^. In catalyst-based OERs, optimal OERs are limited to either acidic, neutral, or alkaline media due to stability issues of the catalyst^11^,^12^. Recently, liquid water-based catalysts were reported for probing water micro-solvation of proteins by water-catalyzed proton-transfer tautomerism and photobiocatalytic chemistry of oxidoreductases using water as the electron donor^13^,^14^. Those results provided new insights into active water’s roles in chemical reactions. In the water-gas shift reaction, Au(111) and Au nanoparticles (AuNPs) have a poor catalytic performance due to their inability to activate one of the most important steps of the reaction, the breaking of O–H bonds during the dissociation of water^15^. We propose that this relatively large energetic barrier can be overcome by utilizing resonantly illuminated AuNPs to facilitate the dissociation of H_2_O. Surface plasmon resonance (SPR) excites the illuminated AuNPs to decay into energetic hot electrons, and instantaneously, hot electron transfer (HET) is utilized to create AuNP-treated with resonant illumination (sAuNT) water with a reduced hydrogen-bonded structure (RHBS)^16^. HET can promote many chemical reactions, including the dissociation of hydrogen^17^. Also, hot electrons can transfer into the MoS_2_ monolayer to induce a structural phase transition^18^. It had been demonstrated that the created sAuNT water exhibited a higher vapor pressure than DI water did due to its weakly hydrogen-bonded structure^16^. This sAuNT water with high chemical potential^19^ would reduce the huge activation energy of OER in conventional DI water. Herein, an innovative strategy on comprehensively efficient oxygen evolution reaction (OER) by utilizing water with reduced hydrogen-bonded structure (RHBS) was proposed. The weak interaction within water molecules significantly lowers the required energy in electrolysis, thus enhancing the efficiency of OER. Also, this advantage was achieved at all pH values.

## Results and Discussion

As shown in Figure S1, the calculated results of degree of the non-hydrogen-bonded structure (DNHBS)^16^ are 21.4% and 26.3% for DI water and sAuNT water, respectively. The increased DNHBS as compared to DI water confirms the presence of created small water clusters on the sAuNT water^16^. Moreover, as shown in Figure S2, in the first hour, the evaporation rate of sAuNT water is increased by ca. 22.6% of magnitude, as compared with that of DI water. Finally, in the 6^th^ hour, this increase has been reduced to ca. 4.7%. This increased evaporation rate as compared to DI water confirms that the created sAuNT water exhibited a higher vapor pressure than DI water did due to its weakly hydrogen-bonded structure.

**Figure 1:**
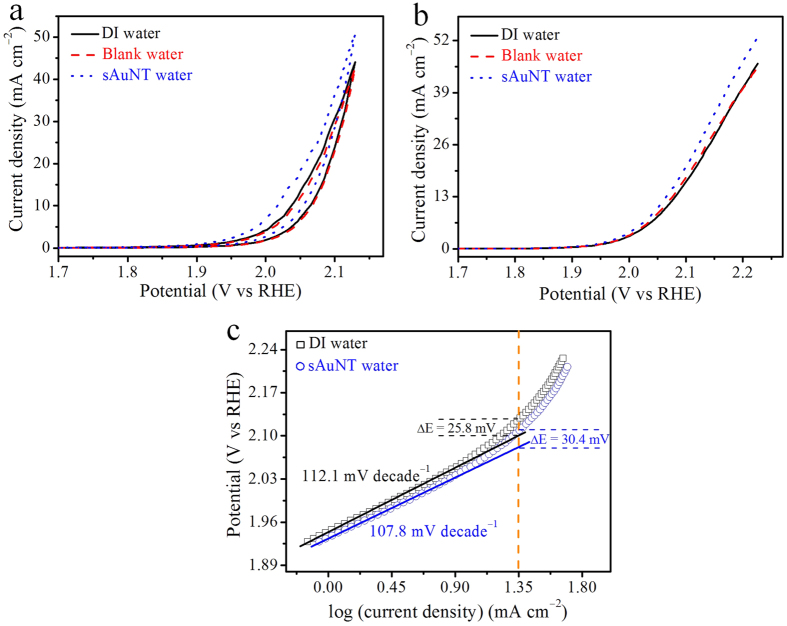
Electrochemical data for oxygen evolution at a planar Pt electrode in various types of water-based solutions with 1 N NaOH as the supporting electrolyte. (**a**) Cyclic voltammograms (CVs) at a scan rate of 50 mV s^−1^ in deionized (DI), blank and gold nanoparticle-treated under resonant illumination (sAuNT) water-based solutions. (**b**) LSV at a scan rate of 50 mV s^−1^ in DI, blank, and sAuNT water-based solutions. (**c**) Comparison of the electroactivity in the oxygen evolution reaction (OER) recorded at the planar Pt electrode in DI water- and sAuNT water-based solutions with 1 N NaOH as the supporting electrolyte.

Figure 1a shows cyclic voltammograms (CVs) of the Pt electrodes in 1 N NaOH solutions based on DI, blank, and sAuNT waters. The blank water was produced according to the process for sAuNT water, but the experiment was performed in a dark atmosphere (light-free). Distinct differences in current densities were observed at the anodic vertex of 2.1 V, in which the current density was 42.7 mA cm^−2^ for the DI water system, and the corresponding current density increased to 50.8 mA cm^−2^ for the sAuNT water system. In addition, the current density and the onset potential (η) based on blank water were almost the same as those for DI water. Correspondingly, the high current density and low η for the sAuNT water system indicate its suitability for application in OERs.

The potential application of sAuNT water in OERs was further evaluated by electrochemical LSV (Fig. 1b). The η for sAuNT water was 1.96 V which was markedly smaller than the 1.99 V for DI water (Figs 1b and S3). This indicates that the required electrolytic energy for the OER was indeed reduced *via* weakening water’s HBs. As the applied potential exceeded the η, the current density distinctly increased and reached a maximum value at 2.2 V. At this vertex, the current density was 52.9 mA cm^−2^ for sAuNT water, which was 14.5% higher than the 46.2 mA cm^−2^ for DI water. Theoretically, the oxidation of water to oxygen is complex science that involves four electrons and four protons forming O–O bonds^20^:


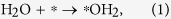



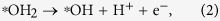



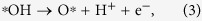



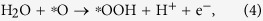


and


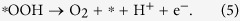


**Figure 2:**
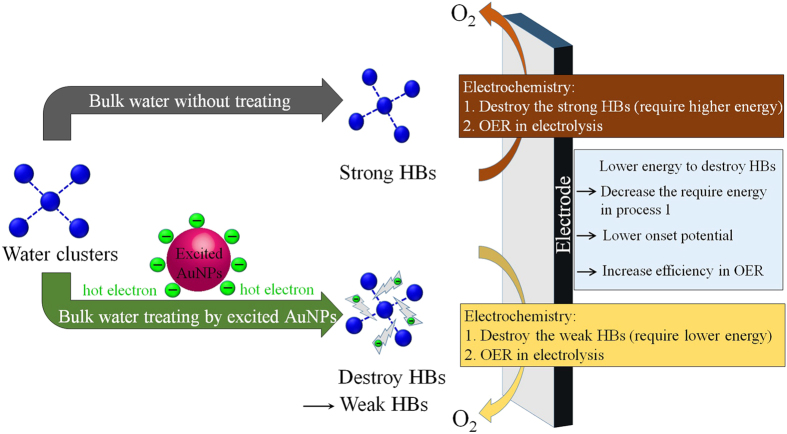
Schematic diagram illustrating the difference of using sAuNT water and DI water in OER. The water with weak hydrogen bond reduces the necessary energy of destroying hydrogen bond within water molecules, thus increasing the efficiency in oxygen evolution reaction.

The lone “*” is a vacant O site of the electrode surface, and *OH_2_, *OH, *O, and *OOH represent the surface with the corresponding chemisorbed species residing in the vacant O site. The electrocatalytic activity of the OER is dependent on the binding energies of *OH_2_, *OH, *O, and *OOH to the electrode surface. To date, exploitation of new electrocatalysts with low η values in the OER has been intensively investigated based on metals and oxide materials^21^,^22^,^23^. However, investigations on reducing η based on the main material “water” in the OER have not been discussed. Water molecules exist in the form of water clusters in bulk water due to strong HB interactions. It was reported that the interaction energy of H_3_O^+^–OH^−^ is 46.9 kJ mol^−1^, and it increases approximately 2.5-times when H_3_O^+^ associates with an additional four water molecules^24^. Based on the two features, the combination of using sAuNT water with weak HBs in OER is proposed (Fig. 2). Firstly, large water clusters associating with strong HBs in bulk water was flowed through the surface of excited AuNPs under resonant illumination; meanwhile the hot electrons were generated from the decay of excited AuNPs, following by surrounding on the surface of AuNPs. The hot electrons would transfer into the bulk water and further destroy the HBs, thus obtaining the sAuNT water with weak HBs. This sAuNT water can lower the require energy for breaking interaction within water molecules before electrochemical dissociation. Therefore, the applied potential for splitting water can be decreased by destroying HBs surrounding each water molecule. Hence, results of a lower η and higher current density for the sAuNT water system in the OER demonstrate that decreasing the degree of the hydrogen-bonded structure may be reflected in the enhanced efficiency of oxygen production. In addition, according to Eq. (1), which describes the adsorption of water molecules onto vacant O sites of the electrode surface, the size of water clusters also dominates the adsorbed quantity of water molecules on the electrode surface. Generally, bulk water clusters with a tetrahedral hydrogen-bonded structure are considered a large size in a normal state. The adsorption process is limited by the steric hindrance due to the large cluster. On the contrary, sAuNT water with reduced HBs means that the original cluster was destroyed to form smaller clusters, resulting in decreased steric hindrance and an increased adsorption quantity. Moreover, similar experiments of Fig. 1b were performed again. The less errors and small differences in experimental data between different batch experiments suggest that the experimental reproducibility is acceptable (See supporting information (SI) and Figure S4).

**Figure 3:**
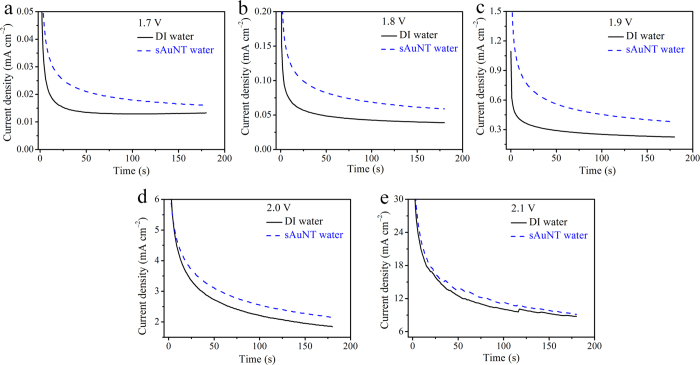
Comparison of current density-time plots at planar Pt electrode in DI water and sAuNT water based solutions with 1 N NaOH as the supporting electrolytes at various applied anodic potentials. (**a**) 1.7 V vs RHE. (**b**) 1.8 V vs RHE. (**c**) 1.9 V vs RHE. (**d**) 2.0 V vs RHE. (**e**) 2.1 V vs RHE.

The relationship between the current density and electrolytic time at different applied potentials from 1.7 to 2.1 V was investigated for DI and sAuNT water systems (Fig. 3). At a lower anodic potential of 1.9 V (Fig. 3a), the current density for DI water quickly reached a stable and extremely low value due to a reduction to its onset η value of 1.99 V; while the current density for sAuNT water took a much longer time to achieve a stable and relatively high value. On the contrary, a small amount of tiny bubbles was generated on the electrode surface which isolated the active site and resulted in the current achieving a steady state with difficulty. At higher applied potentials (Fig. 3b–e), the generated bubbles absorbed onto the electrode led to the recorded current density gradually decreasing with electrolytic time, especially in the sAuNT water system. This suggests that a longer time to record a stable current density may represent a more-efficient OER (see SI).

The kinetics of the OER were evaluated by the Tafel slope which describes the influence of the potential or η on the steady-state current density^25^. Tafel plots of DI and sAuNT water-based systems on the OER are shown in Fig. 1c. At lower η values, the Tafel slope based on sAuNT water (107.8 mV decade^−1^) was smaller at 4.3 mV decade^−1^ than that based on DI water (112.1 mV decade^−1^), indicating that sAuNT water possesses better catalytic activity in the OER. At higher η values, the polarization curve based on sAuNT water deviated from the linear region at about 30.4 mV, which was larger than the 25.8 mV for DI water by 4.6 mV. The deviation in the polarization curve from the linear region was due to the generation of oxygen, which decreases the active surface area^26^. Thus, this larger deviation of the polarization curve at the higher η observed with the sAuNT water-based electrolyte again indicates its higher catalytic activity in the OER.

**Figure 4:**
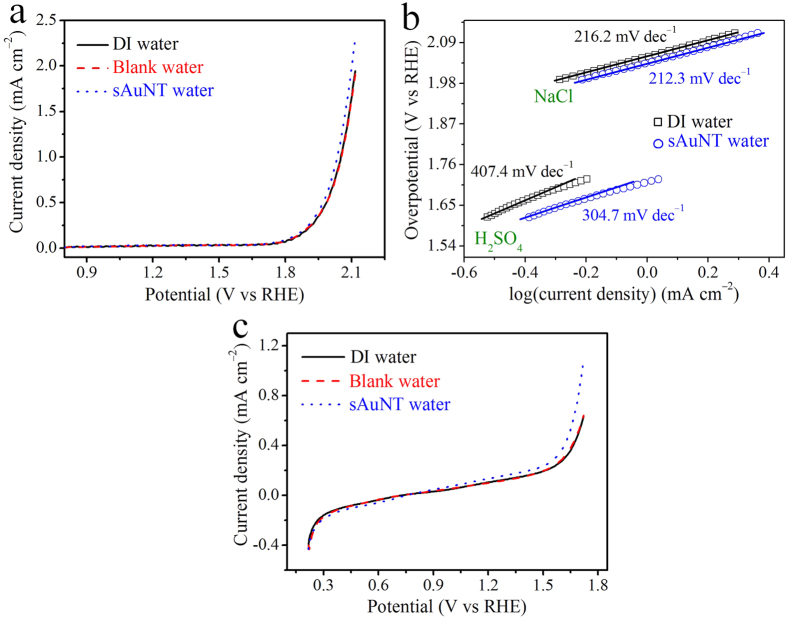
Performance and electroactivity of the oxygen evolution reaction (OER) at a planar Pt electrode in various types of water-based neutral and acidic electrolytes. (**a**) LSV in deionized (DI) water-, blank water-, and gold nanoparticle-treated under resonant illumination (sAuNT) water-based neutral electrolytes (0.1 N KCl). (**b**) The corresponding Tafel plots of DI water- and sAuNT water-based neutral (0.1 N KCl) and acidic (1 N H_2_SO_4_) electrolytes. (**c**) LSV in DI water-, blank water- and sAuNT water-based acidic electrolytes (1 N H_2_SO_4_).

The enhanced efficiencies of the OER in the sAuNT water system were also evaluated in neutral and acidic electrolytes. In the neutral electrolyte, the recorded current density at 2.1 V in the sAuNT water-based solution was 2.3 mA cm^−2^, which was 21.1% higher than that in the DI water-based solution (1.9 mA cm^−2^) (Fig. 4a). The onset η in sAuNT water was 1.90 V, which was lower than the 1.95 V in DI water. In addition, the Tafel slope of linear fitting at the lower η for sAuNT water was 212.3 mV decade^−1^, which was smaller than the 216.2 mV decade^−1^ for DI water (Fig. 4b). Similar to the results shown with alkaline and neutral electrolytes, the OER based on sAuNT water exhibited a higher current density (an increase of 69.3%), a lower onset η, and a lower Tafel slope in acidic electrolyte (Fig. 4b,c). These results suggest that the enhanced efficiency of the OER when using sAuNT water instead of conventional DI water is suitable at all pH values.

**Figure 5:**
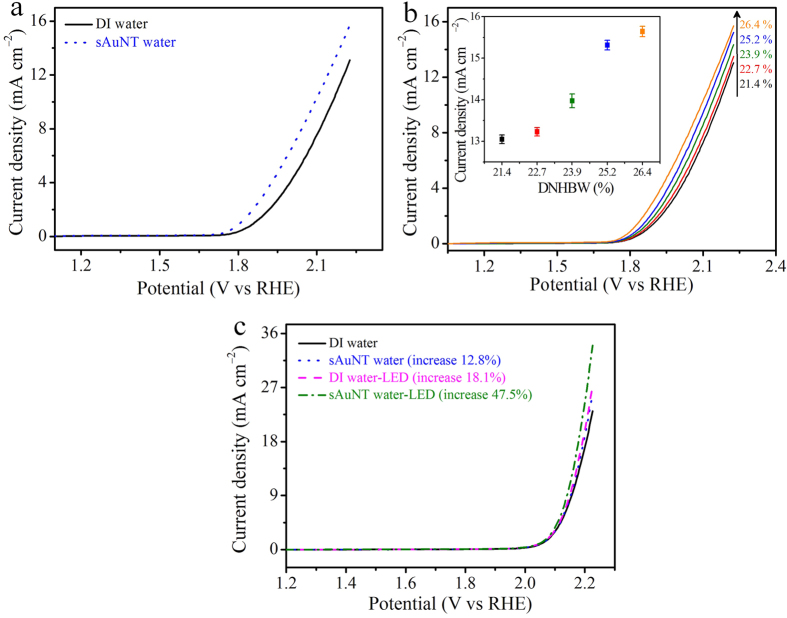
Effect of the degree of non-hydrogen-bonded water (DNHBW) on the efficiency of the oxygen evolution reaction (OER) at an indium tin oxide (ITO) electrode in 1 N NaOH and its *in situ* experiment based on a roughened Au electrode. (**a**) LSV for the OER in deionized (DI) water- and gold nanoparticle-treated under resonant illumination (sAuNT) water-based solutions. (**b**) Effect of DNHBW of water on the increased current density in the OER. (**c**) Experiments of *in situ* breaking of hydrogen bonds of water combining the OER at an electrochemically roughened Au electrode in alkaline electrolyte of 1 N NaOH. LSV for DI (solid line) and sAuNT (dot) water-based systems on the roughened Au electrode in the dark without illumination. LSV for DI (dash) and sAuNT (dash dot) water-based systems on a roughened Au electrode under resonant illumination.

To evaluate the effect of sAuNT water at the electrode on the efficiency of the OER, the Pt electrode was replaced by an inert indium tin oxide (ITO) electrode (0.28 cm^2^) based on an alkaline electrolyte (Fig. 5a). The current density at 2.2 V was 15.7 mA cm^−2^ for sAuNT water, which was still higher than the 13.1 mA cm^−2^ for DI water (an increase of 19.8%). This increase was higher than that observed with the Pt electrode (14.5%). This reveals that the Pt electrode was also contributive to the catalytic OER, and thus a decrease in the contribution from sAuNT water to the total efficiency of the OER was observed with the Pt electrode. Furthermore, the efficiency of OER was tunable by adjusting degree of non-hydrogen-bonded water (DNHBW) (Fig. 5b).

Furthermore, the *in situ* reduction of water’s HBs combined with the OER was performed on the AuNP-deposited Au electrode under illumination in an alkaline electrolyte to further clarify this innovative idea of efficient OERs (Fig. 5c). It can be observed that the current density at 2.2 V increased 12.8% in sAuNT water compared to DI water in the dark. This means that utilizing sAuNT water to improve the efficiency of the OER was also suitable with the roughened Au electrode. In addition, the onset η of the OER was more negative when the roughened Au electrode was illuminated with a green LED. The current density for the OER at 2.2 V significantly increased 18.1% due to the synchronous breaking of HBs within water molecules during electrocatalysis. This increase was further enhanced to 47.5% when DI water was replaced with sAuNT water. This increase was higher than the increase summarized from the experiment performed in sAuNT water in the dark (12.8%) and that performed in DI water under LED illumination. This suggests a synergistic effect indeed occurred when electrocatalyzing sAuNT water on an AuNP-deposited Au electrode under resonant illumination.

**Figure 6:**
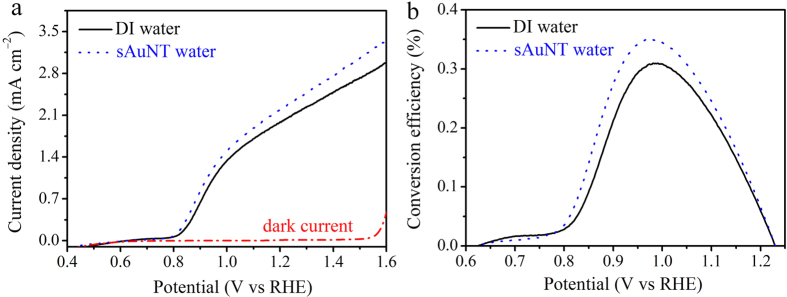
Effect of sAuNT water on the efficiency of the oxygen evolution reaction (OER) at a Co-Pi@ZnFe_2_O_4_@α-Fe_2_O_3_:Ti photoelectrode in 1 N NaOH. (**a**) Photocurrent density-potential (J–V) curves and (**b**) efficiencies of the hematite photoelectrodes in DI and sAuNT water electrolytes. The illumination conditions were AM 1.5 G and 100 mW cm^−2^. Dark current was recorded in DI water in a dark atmosphere.

To demonstrate the water oxidation ability of sAuNT water compared with DI water, Co-Pi oxygen evolution catalysts decorated hematite (Co-Pi@ZnFe_2_O_4_@α-Fe_2_O_3_:Ti) nanomaterials were employed as photoelectrodes for photocurrent measurement in a three-electrode electrochemical system with simulated solar illumination (AM 1.5 G 100 mW cm^−2^), as shown in Fig. 6a. Accordingly, we could observe that the water oxidation photocurrents in both cases began at the same potential (0.62 V vs RHE); however, the photocurrent density at 1.23 V vs RHE of hematite photoelectrode in the sAuNT water electrolyte could achieve 2.3 mA cm^−2^, which was higher than that in DI water electrolyte (2.0 mA cm^−2^). In addition, the efficiencies of hematite photoelectrodes^27^ in DI and sAuNT water estimated by Eq. (6) were 0.31% and 0.35%, as shown in Fig. 6b.


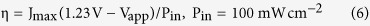


From above photocurrent measurement, we could find that the photogenerated holes are easily transferred from hematite photoelectrode into electrolyte when sAuNT water was used as solvent. We speculate that sAuNT water owns high water oxidation ability due to large potential difference between valence band of hematite and water oxidation potential (E_ox_(H_2_O/O_2_)). Therefore, the photogenerated holes transfer resistance in sAuNT water is lower than that in DI water.

In addition, the metal oxides such as iron oxide and its complex have been generally considered as good candidates as OER catalysts^28^,^29^. Therefore, compared to the Pt and ITO electrodes which belong to the inert catalysts for OER, the more active catalyst of iron oxide (Fe_3_O_4_) is examined for OER performed in DI water-based and sAuNT water-based solutions with 1 N NaOH. As shown in Figure S5, the onset potential in DI water-based and sAuNT water-based solutions were 1.80 V and 1.76 V. Also, the current density at 2.0 V was 44.3 mA cm^−2^ for DI water-based solution and 58.2 mA cm^−2^ for sAuNT water-based solution (a significant increase of 31.4%). Examining the onset potential or the current density, the Fe_3_O_4_-modified Au electrode showed a better performance in OER as compared to Pt and ITO electrodes. It indicates that Fe_3_O_4_ indeed owns a better catalytic activity for OER. Conclusively, these results reveal the enhanced OERs by using sAuNT water-based solutions are effective for active and inert catalysts.

In summary, we have innovatively utilized sAuNT water with weak hydrogen bonds in an efficient OER on a catalytic, inert or photosensitive electrode. The lower onset η and higher current density based on the sAuNT water system revealed that it is favorable for promoting efficient oxygen production. Moreover, utilizing sAuNT water with increasing electroactivity of the OER was suitable at all pH values. Different from conventional explorations for new active materials to modify electrodes, this study establishes an entirely new viewpoint on the efficiency of the OER associated with the strength of interactions within water molecules. It is expected that an outstanding performance of the OER will be realized by synergistic effects between sAuNT water and highly catalytically active electrodes.

### Methods summary

The preparation of sAuNT water was treating DI water by excited AuNPs. The efficiency of OER was measured by power potential.

Full Methods and any associated references are available in the online version of the paper.

## Additional Information

**How to cite this article**: Chen, H.-C. *et al.* Triggering comprehensive enhancement in oxygen evolution reaction by using newly created solvent. *Sci. Rep.* **6**, 28456; doi: 10.1038/srep28456 (2016).

## Supplementary Material

Supplementary Information
