# Supplementary material for: Triggering comprehensive enhancement in oxygen evolution reaction by using newly created solvent
Source: Sci Rep. 2016 Jun 22;6:28456. doi: 10.1038/srep28456 (PMC4916470; doi:10.1038/srep28456)
Supplement: Supplementary Information [file srep28456-s1.doc]

**Supplementary Information**

**Triggering comprehensive enhancement in oxygen evolution reaction by using newly created solvent**

Hsiao-Chien Chen^1^, Fu-Der Mai^1^, Kuang-Hsuan Yang^2^, Liang-Yih Chen^3^, Chih-Ping Yang^4^, Yu-Chuan Liu^1,*^

^1^ Department of Biochemistry and Molecular Cell Biology, School of Medicine, College of Medicine, Taipei Medical University, No. 250, Wuxing St., Taipei 11031, Taiwan.

^2^ Department of Materials Science and Engineering, Vanung University, No. 1, Van Nung Rd., Chung-Li City, Taiwan.

^3^ Department of Chemical Engineering, National Taiwan University of Science and Technology, No. 43, Sec. 4, Keelung Rd., Taipei 10607, Taiwan.

^4^ Graduate Institute of Medical Science, College of Medicine, Taipei Medical University, No. 250, Wuxing St., Taipei 11031, Taiwan.

Correspondence and requests for materials should be addressed to Y.-C. Liu, (E-mail: [liuyc@tmu.edu.tw](mailto:liuyc@tmu.edu.tw)).

**Supplementary Methods**

**Chemicals and materials.** Sulfuric acid sodium, potassium chloride and hydroquinone were purchased from Sigma-Aldrich Organics. 40 mesh-screened ceramic particles (Molar compositions: 92 % SiO_2_, 3.0 % Na_2_O and K_2_O, 2.0 % Fe_2_O_3_, 1.5 % Al_2_O_3_, 0.5 % CaO, 0.5 % MgO, and other rare metal oxides) for filtering through deionized (DI) water were purchased from Chyuan-Bang enterprise Co., Ltd., Taiwan. Commercial chitosan (Ch) powders with a degree of deacetylation of 0.82 were purchased from First Chemical Works, Taiwan. All of the solutions were prepared using deionized (DI) water (18.2 MΩ cm) provided from a Milli-Q system. All of the experiments were performed in an air-conditioned room at ca. 24 ^o^C. The water temperature is ca. 23.5 ^o^C.

**Preparation of ceramic particles-supported Au NPs.** The rinsed ceramic particles were immersed in a solution containing 30 ppm AuNPs for 1 day. Then the Au NPs-adsorbed ceramic particles were rinsed throughout with deionized water, and finally dried in an oven at 100 ^o^C for 1 day. Subsequently, the prepared Au NPs-adsorbed ceramic particles were loaded in a valve-equipped glass tube (I.D.: 30 mm, L: 300 mm). Before prepare the reduced hydrogen-bonded liquid water the ceramic particles-supported AuNPs in the glass tube were rinsed with deionized water for several cycles until the pH values of DI water before and after it passed through the particle-loaded tube are almost identical (ca. pH 7.23 and water temp. at ca. 23.5 ^o^C).

**Preparation of AuNT, AuNT (light-free), and sAuNT waters.** For these preparations, DI water (pH 7.23, T=23.5 °C) was passed through a glass tube filled with AuNP-adsorbed ceramic particles under fluorescent lamp illumination. Then the AuNT water (pH 7.25, T=23.3 °C) was immediately collected in glass sample bottles for subsequent tests. To further examine the purity of the prepared AuNT water, inductively coupled plasma-mass spectrometric (ICP-MS) analyses indicated that the concentrations of the slightly dissolved metals in the AuNT water were ca. 0.62, 43, 25, 23, 13, 4.5, and 0.41 ppb for Au, Na, K, Al, Mg, Ca, and Fe, respectively. Excluding Au, the total equivalent molar concentration of these dissolved metals was equal to ca. 6.9×10^-6^ N. This measured value is ca. 2.4×10^-7^ N for DI water as a reference. Similarly, the AuNT water (light-free) was produced according to the above processes in a dark room without illumination. sAuNT water was also produced according to the above processed but under illumination with green light (532 nm) of light-emitting diode (LED).

**Oxygen evolution reaction (OER) measurements.** Typically, the oxygen evolution reaction was performed by a cyclic or linear sweep voltammetry in a three-electrode system consisting of a Pt electrode (0.07 cm^2^), a Pt sheet and an Ag/AgCl as the working, counter and reference electrodes, respectively. The corresponding electrochemical measurement was performed in 50 mL deoxygenated solution with 1 N NaOH (or 0.1 N KCl, or 1 N H_2_SO_4_) at a scan rate of 0.05 V s^–1^.

**Preparation of gold nanoparticles.** The AuNPs in an aqueous solution was obtained from an Au sheet (purity of 0.9999) by using electrochemical and thermal reduction methods, as shown in our previous report^30^. Typically, the Au electrode was cycled in a deoxygenated aqueous solution of 40 mL containing 0.1 M NaCl and 1 g L^-1^ Ch from -0.28 to +1.22 V vs Ag/AgCl at 500 mV s^-1^ for 200 scans under slight stirring. The durations at the cathodic and anodic vertices are 10 and 5 s, respectively. Immediately, without changing the electrolytes, the solution was heated from room temperature to boiling at a heating rate of 6 ^o^C min^-1^ in air. After cooling the clear Au NPs-containing solution was separated from the settlement of Ch. Then the AuNPs-containing solution was placed in an ultrasonic bath for 30 min and was further centrifugalized at 3600 rpm for 2 min to remove Ch for preparing pure AuNPs in solution.

**Preparation of Co-Pi@ZnFe_2_O_4_@α-Fe_2_O_3_:Ti/FTO electrode.** A heterostructured Co-Pi@ZnFe_2_O_4_@α-Fe_2_O_3_:Ti nanomaterials onto fluorine-doped tin oxide (FTO) substrate was prepared as photoelectrodes. α-Fe_2_O_3_ nanomaterials was synthesized by an aqueous solution (19.5 mL) containing 75 mM FeCl_3_·6H_2_O (Alfa Aesar, 98%), 0.5 M NaNO_3_ (J. T. Baker, ACS reagent) and 0.5 mL of ethanol solution at pH 1.5 (adjusted by HCl). The growth solution was poured into an autoclave with Teflon liner. FTO substrate was placed in the liner with the FTO side facing the wall of the liner and heated at 100^o^C for 4 h. After the reaction, the film synthesized on FTO substrate was thoroughly rinsed with deionized water and annealed at 500^o^C for 1 h in air to obtain the desired phase (α-Fe_2_O_3_). For forming titanium doped α-Fe_2_O_3_ (α-Fe_2_O_3_:Ti) photoelectrode, 2.5 vol% TiCl_4_ ethanol solution was prepared. Then drop-coating process was employed onto the surface of α-Fe_2_O_3_ photoelectrode. After drop-coating, the photoelectrode was rinsed with ethanol solution and annealed at 650^o^C for 2 h in N_2_. ZnFe_2_O_4_(ZFO)@α-Fe_2_O_3_:Ti heterostructured photoelectrode was prepared by dropping 3 times of 20 μL ethanol solution of 5 mM Zn(OAc)_2_∙2H_2_O onto the α-Fe_2_O_3_:Ti photoelectrode. The resulting sample was rinsed by ethanol and annealed at 500^o^C for 30 min in N_2_. Co-Pi was photoelectrodeposited on hematite photoelectrodes as oxygen evolution catalyst (OEC). A three-electrode setup was employed with hematite as the working electrode, Ag/AgCl (3 M KCl) as the reference electrode and Pt foil as the counter electrode. The electrolyte solution containing 0.5 mM CoCl_2_ (Alfa Aesar, 97%) in 1 M KH_2_PO_4_ (J. T. Baker, ACS reagent) aqueous solution adjusted to pH 7. The working electrode was biased to 0.4 V vs Ag/AgCl, controlled by a PGSTAT 302N potentiostate (Metrohm Autolab) under 302 nm illumination for 10 min.

Photocurrent density measurement was conducted in the electrolyte solution of 1 N NaOH aqueous solution (pH=13.6) under a xenon lamp (Newport 6258, 300 W) with an AM 1.5 G filter to provide a light intensity of 100 mW/cm^2^. The incident light intensity was calibration by a standard crystalline silicon solar cell (Oriel reference cell, 91550V). Pt foil and Ag/AgCl (3 N KCl) (E^o^_Ag/AgCl_ = 0.21 V vs NHE at 25^o^C) were counter electrode and reference electrode, respectively. The scan range was at 0.4 V~1.6 V and the scan rate was 10 mV sec^–1^. The working area of hematite nanomaterial photoanode was 0.16 cm^2^. According to Nernst equation^31,32^, the RHE potential is defined as follows.

$E_{\text{RHE}}=E_{\text{Ag/AgCl}}+0.0591\cdot\text{pH}+E_{\text{Ag/AgCl}}^{o}$ (1)

where E_RHE_ is the converted potential vs RHE, E_Ag/AgCl_ is the experimentally measured potential against the Ag/AgCl (3 N KCl) reference electrode. The efficiency was determined by the following equation.^27^

η = J_max_ (1.23 V-V_app_)/P_in_, P_in_ = 100 mW cm^–2^ (2)

Where J_max_ is photocurrent density in highest STH conversion efficiency, V_app_ is applied voltage from cathodic current to anodic current, and P_in_ is the power density of solar illumination. IPCE were measured using a Xenon lamp (Oriel arc lamp #66160, 300W) coupled with a monochromator (Oriel Cornerstone 130 1/8m).

**Preparation of iron oxide (Fe_3_O_4_).** The preparation of magnetic Fe_3_O_4_ NPs was according to the previous literature^33^. Typically, 2.16 mmol FeCl_3_ and 3.24 mmol FeCl_2_·4H_2_O were dissolved in DI water (200 mL). The temperature was raised to 60 ^o^C and then 0.864 N NaOH (10 mL) was added slowly into the solution as the temperature was further raised to 80 ^o^C within 5 min. Finally, the reaction cell was immersed into ice-water bath and the prepared Fe_3_O_4_ NPs was collected by magnet. The collected Fe_3_O_4_ was re-dispersed into 10 mL DI water.

**Preparation of Fe_3_O_4_-modified Au electrode.** First, a magnet was fixed at the back of Au electrode. Then, 10 µL of magnetic Fe_3_O_4_ solution was dropped and was adsorbed magnetically onto the bare circle (surface area of 0.28 cm^2^) on the Au electrode.

**Supplementary Discussion**

**1.** **LSV of sAuNT water in different applied potentials**

When referenced to the DI water system, the efficiency of the OER at 180 s for the sAuNT water system increased from 23.1% to 51.3% and 67.6% as the applied potential increased from 1.7 to 1.8 and 1.9 V vs RHE, respectively. However, values dramatically dropped to 16.3% and 4.5% as the applied potential increased to 2.0 and 2.1 V vs RHE, respectively. At the applied potential close to the onset η, the degree of hydrogen-bonded structure in water dominates the entire efficiency of the OER. As the applied potential further anodically increased, the required energy to break hydrogen-bonded structures was no longer significant under a sufficiently applied energy.

**2.** **The effect of DNHBS of water to efficiency of OER**

The degree of the non-hydrogen bonded structure (DNHBS) of water can be easily adjusted by mixing sAuNT and DI waters in different ratios. As shown in Figure 4b, the current density increased from 13.1 mA cm^–2^ to 13.3, 14.0, 15.3, and 15.7 mA cm^–2^ as the DNHBS increased from 21.4% (for DI water) to 22.7%, 23.9%, 25.2%, and 26.4% (for sAuNT water), respectively. Obviously, the current density can be significantly increased with an increase in the DNHBS of water. Although it did not display a linear relationship, it truly demonstrates again that water molecules with weak hydrogen bonds are favorable for an efficient OER.

**3.** **Reproducibility of OER**

In this work, good data reproducibility had already been confirmed in Figure 4c in the text, regarding effect of the degree of non-hydrogen bonded structure (DNHBS) in water on the efficiency of OER at an ITO electrode in 1 N NaOH. Moreover, similar experiments of Fig. 1b are performed again, as shown in Figure S2, including sAuNT water* sample. It represents sAuNT water was cooled at 4 ^o^C for 6 h and placed in laboratory at room temperature to raise its temperature naturally to the room temperature before OER experiment. At the vertex of 2.2 V vs RHE, the current density is 51.6±1.01 mA cm^–2^ (52.9 mA cm^–2^ reported in the original text) for sAuNT water, which is 12.9% (14.5% reported in the original text) higher than the 45.7±0.71 mA cm^–2^ (46.2 mA cm^–2^ reported in the original text) for DI water. The less errors and small differences in experimental data between different batch experiments suggest that the experimental reproducibility is acceptable. In addition, the significant differences of recorded current densities for experiments performed in different waters of sAuNT water, sAuNT water* and DI water suggest that the impurity in sAuNT influences less on the significantly increased OER efficiency in sAuNT water-based solution.

**Figure S1**


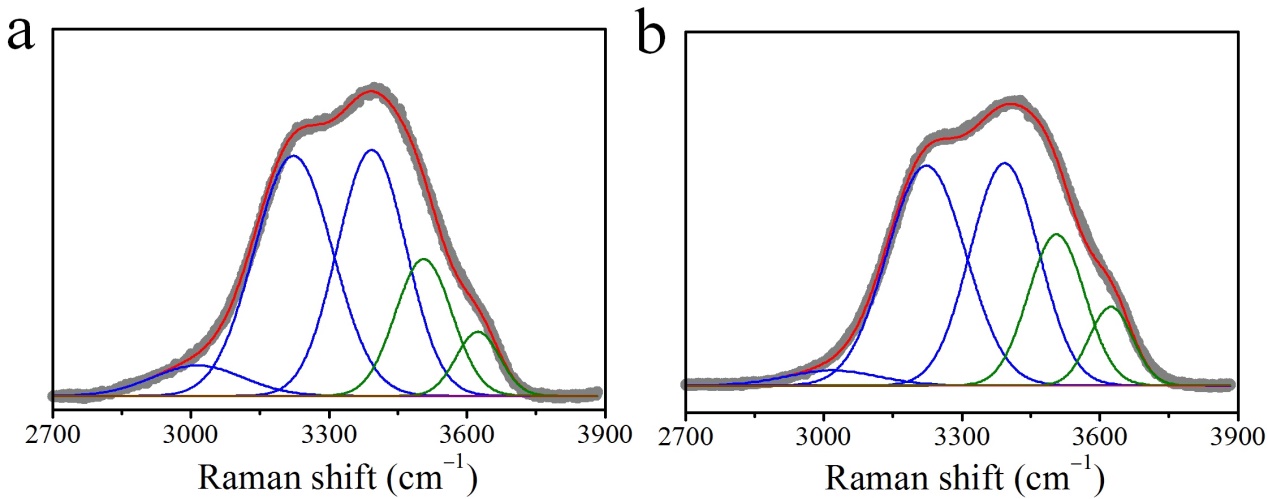


**Figure S1.** Raman spectra of (a) DI water (b) sAuNT water. According to our previous report^16^, the Raman spectra of water within the range from 2700 to 3900 cm^–1^ could be deconvoluted into five-Gaussian components with center wavenumbers of 3018, 3223, 3393, 3506 and 3624 cm^–1^. Moreover, the full width at half maximum (FWHM) to each sample are 234, 201, 176, 154 and 112 cm^–1^, respectively. The degree of the non-hydrogen-bonded structure (DNHBS) in water is defined as the ratio of the area of the non-hydrogen-bonded OH stretching bands (at 3506 and 3624 cm^–1^) to the areas of the total stretching bands.

**Figure S2**

**Figure S2.** Evaporation rates (g h^-1^) of DI water (black block) and sAuNT water (blue block) at 1 atm and room temperature. In experiment, each 50 mL of DI water and sAuNT water were individually added in glass bottles (70 mL) which were placed on the same orbital shaker at 50 rpm. The weights of each bottle containing water were measured every hour for 6 h to calculate the weight losses of DI water and sAuNT water during evaporation.

**Figure S3**


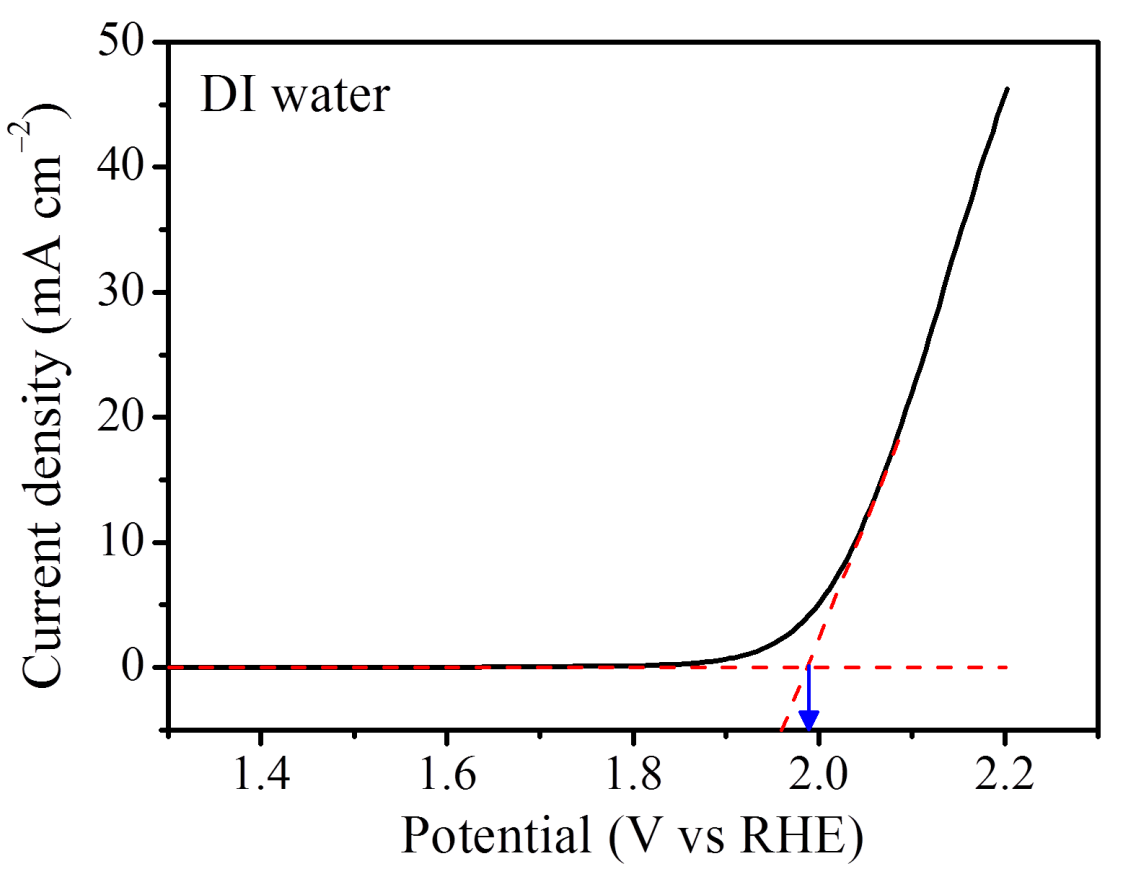


**Figure S3. Criterion method for determining the onset potential in OER.** Figure 1b in the text is shown for an example. First, we plotted two tangent lines on this I-V curve. Then, a cross point was obtained. The corresponding potential based on this cross point is defined as the onset potential. In this case, the obtained onset potential is 1.99 V for DI water.

**Figure S4**

**
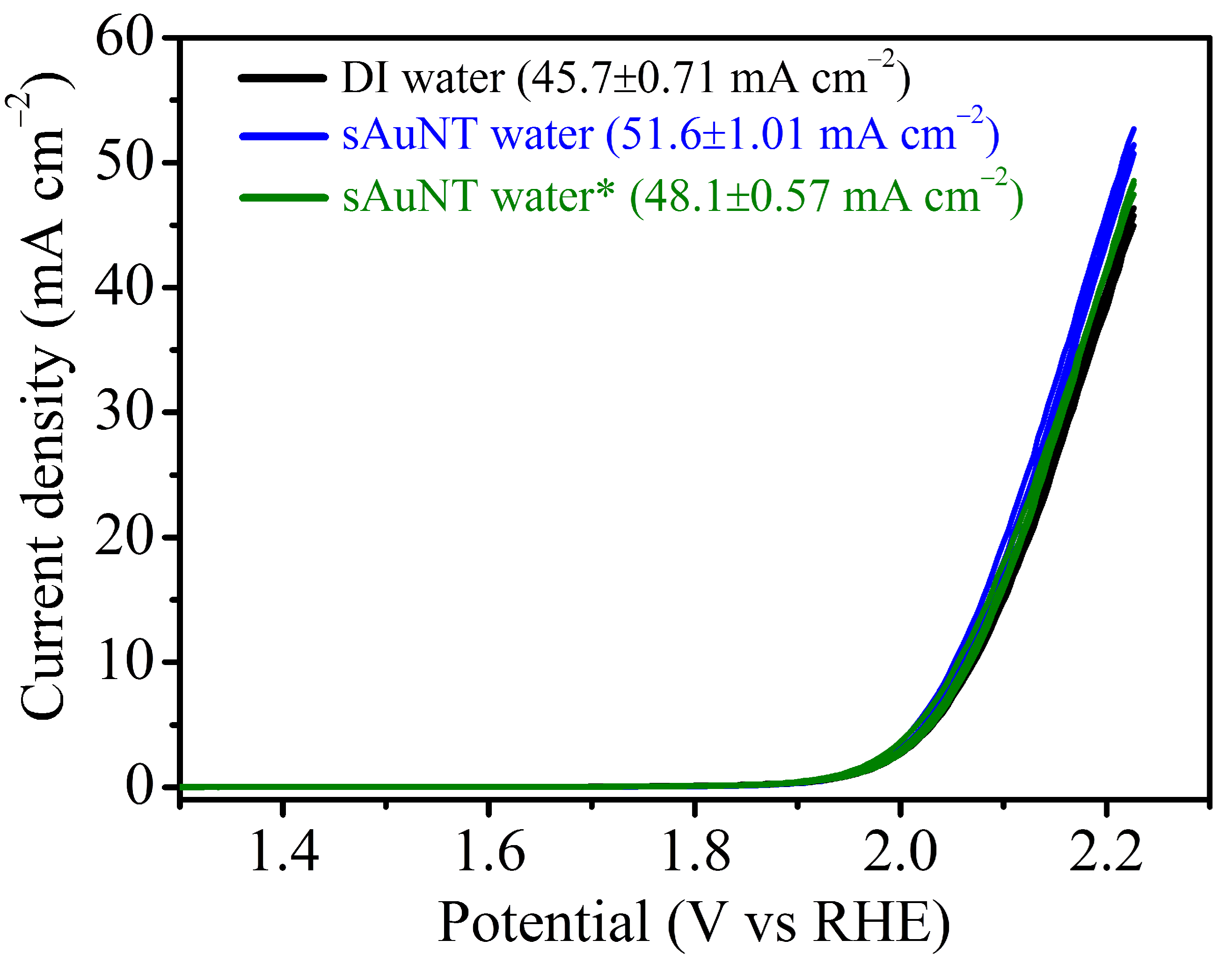
**

**Figure S4.** Electrochemical data for oxygen evolution at a planar Pt electrode in various types of water-based solutions with 1 N NaOH as the supporting electrolyte, showing LSV at a scan rate of 50 mV s^-1^ in DI, blank, and sAuNT water-based solutions. sAuNT water* represents sAuNT water was cooled at 4 ^o^C for 6 h and placed in laboratory at room temperature to raise its temperature naturally to the room temperature before experiment.

**Figure S5**

**Figure S5.** LSV of Fe_3_O_4_-modified Au electrode at a scan rate of 50 mV s^-1^ in DI water-based and sAuNT water-based solutions with 1 N NaOH.

**References**

1. Laage, D., Stirnemann, G., Sterpone, F. & Hynes, J. T. Water jump reorientation: from theoretical prediction to experimental observation. *Accounts Chem. Res*. **45**, 53-62 (2012).

2. Laage, D. & Hynes, J. T. A molecular jump mechanism of water reorientation. *Science* **311**, 832-835 (2006).

3. Pamasesha, K., Marco, L. D., Mandal, A. & Tokmakoff, A. Water vibrations have strongly mixed intra- and intermolecular character. *Nat. Chem.* **5**, 935-940 (2013).

4. Zhang, J., Chen, P., Yuan, B., Ji, W., Cheng, Z. & Qiu, X. Real-space identification of intermolecular bonding with atomic force microscopy. *Science* **342**, 611-614 (2013).

5. Li, R., Jiang, Z., Guan, Y., Yang, H. & Liu, B. Effects of metal ion on the water structure studied by the Raman O-H stretching spectrum. *J. Raman Spectrosc*. **40**, 1200-1204 (2009).

6. Sun, B., Huang, X., Chen, S., Munroe, P. & Wang, G. Porous graphene nanoarchitectures: an efficient catalyst for low charge-overpotential, long life, and high capacity lithium–oxygen batteries. *Nano Lett.* **14**, 3145-3152 (2014).

7. Bergmann, A., Zaharieva, I., Dau, H. & Strasser, P. Electrochemical water splitting by layered and 3D cross-linked manganese oxides: correlating structural motifs and catalytic activity. *Energ. Environ. Sci.* **6**, 2745-2755 (2013).

8. Song, F. & Hu, X. Exfoliation of layered double hydroxides for enhanced oxygen evolution catalysis. *Nat. Commun.* **5**, 4477 (2014).

9. Suntivich, J., May, K. J., Gasteiger, H. A., Goodenough J. B. & Shao-Horn, Y. A Perovskite oxide optimized for oxygen evolution catalysis from molecular orbital principles. *Science* **334**, 1383-1385 (2011).

10. Louie, M. W. & Bell, A. T. An investigation of thin-film Ni–Fe oxide catalysts for the electrochemical evolution of oxygen. *J. Am. Chem. Soc.* **135***,* 12329-12337 (2013).

11. Smith, R. D. L., Sporinova, B., Fagan, R. D., Trudel, S. & Berlinguette, C. P. Facile photochemical preparation of amorphous iridium oxide films for water oxidation catalysis. *Chem. Mater.* **26***,* 1654-1659 (2014).

12. Kanan, M. W. & Nocera, D. G. In situ formation of an oxygen-evolving catalyst in neutral water containing phosphate and Co^2+^. *Science* **321**, 1072-1075 (2008).

13. Shen, J. Y. *et al.* Probing water micro-solvation in proteins by water catalyzed proton-transfer tautomerism. *Nat. Commun.* **4**, 2611 (2013).

14. Mifsud, M., Gargiulo, S., Iborra, S., Arends, I. W., Hollmann, F. & Corma, A. Photobiocatalytic chemistry of oxidoreductases using water as the electron donor. *Nat. Commun.* **5**, 3145 (2014).

15. Rodriguez, J. A., Senanayake, S. D., Stacchiola, D., Liu, P. & Hrbek, J. The activation of gold and the water–gas shift reaction: insights from studies with model catalysts. *Accounts Chem. Res*. **47**, 773-782 (2014).

16. Chen, H. C. *et al.* Active and stable liquid water innovatively prepared using resonantly illuminated gold nanoparticles. *ACS Nano* **8**, 2704-2713 (2014).

17. Mukherjee, S. *et al.* Hot electrons do the impossible: plasmon-induced dissociation of H_2_ on Au. *Nano Lett.* **13**, 240-247 (2013).

18. Kang, Y. *et al.* Plasmonic hot electron induced structural phase transition in a MoS_2_ monolayer. *Adv. Mater.* **26**, 6467-6471 (2014).

19. van den Berg, C. & Bruin, S. Water activity and its estimation in food systems: theoretical aspects. In: ROCKLAND, L. B.; STEWART, G. F. (Eds.). Water Activity: Influences on Food Quality. New York: Academic Press, 1981.

20. Liao, P., Keith, J. A. & Carter, E. A. Water oxidation on pure and doped hematite (0001) surfaces: prediction of Co and Ni as effective dopants for photocatalysis. *J. Am. Chem. Soc.* **134**, 13296-13309 (2012).

21. Zhuang, Z., Sheng, W. & Yan, Y. Synthesis of monodispere Au@Co_3_O_4_ core-shell nanocrystals and their enhanced catalytic activity for oxygen evolution reaction. *Adv. Mater.* **26**, 3950-3955 (2014).

22. Lu, Z. *et al.* Electrochemical tuning of layered lithium transition metal oxides for improvement of oxygen evolution reaction. *Nat. Commun.* **5**, 5345 (2014).

23. Gao, M. R. *et al.* Nitrogen-doped graphene supported CoSe_2_ nanobelt composite catalyst for efficient water oxidation. *ACS Nano* **8**, 3970-3978 (2014).

24. Lee, H. M. & Kim, K. S. Dynamics and structural changes of small water clusters on ionization. *J. Comput. Chem.* **34**, 1589-1597 (2013).

25. Long, X. *et al.* A strongly coupled graphene and FeNi double hydroxide hybrid as an excellent electrocatalyst for the oxygen evolution reaction. *Angew. Chem. Int. Ed.* **53**, 7584-7588 (2014).

26. Li, Y. G., Hasin, P. & Wu, Y. Y. NixCo_3_−xO_4_ nanowire arrays for electrocatalytic oxygen evolution. *Adv. Mater.* **22**, 1926-1929 (2010).

27. Walter, M. G. *et al.* Solar water splitting cells. *Chem. Rev.* **110**, 6446-6473 (2010).

28. Kurzman, J. A., Dettelbach, K. E., Martinolich, A. J., Berlinguette, C. P. & Neilson, J. R. Structural characteristics and eutaxy in the photo-deposited amorphous iron oxide oxygen evolution catalyst. *Chem. Mater.* **27**, 3462-3470 (2015).

29. Chen, M. *et al.* An iron-based film for highly efficient electrocatalytic oxygen evolution from neutral aqueous solution. *ACS Appl. Mater. Interfaces* 7**,** 21852-21859 (2015).

30. Yu, C. C., Liu, Y. C., Yang, K. H. & Tsai, H. Y. Simple method to prepare size-controllable gold nanoparticles in solutions and their applications on surface-enhanced Raman scattering. *J. Raman Spectrosc*. **42**, 621-625 (2011).

31. Zhong, D. K., Cornuz, M., Sivula, K., Grätzel M. & Gamelin, D. R. Photo-assisted electrodeposition of cobalt–phosphate (Co–Pi) catalyst on hematite photoanodes for solar water oxidation. *Energ. Environ. Sci.* **4**, 1759-1764 (2011).

32. Barroso, M., Cowan, A. J., Pendlebury, S. R., Grätzel, M., Klug, D. R. & Durrant, J. R. The role of cobalt-phosphate in enhancing the photocatalytic activity of α-Fe_2_O_3_ toward water oxidation. *J. Am. Chem. Soc.* **133**, 14868-14871 (2011).

33. Chen, H. C. *et al.* Magnetic-composite-modified polycrystalline silicon nanowire field-effect transistor for vascular endothelial growth factor detection and cancer diagnosis. *Anal. Chem.* **86**, 9443-9450 (2014).
